# Innovation and The Israel Journal of Health Policy Research

**DOI:** 10.1186/s13584-019-0353-1

**Published:** 2019-12-03

**Authors:** Bruce Rosen, Avi Israeli, Stephen Schoenbaum

**Affiliations:** 1 Myers-JDC-Brookdale Institute, JDC Hill, Jerusalem, Israel; 2 The Hebrew University - Hadassah Medical Center-, Jerusalem, Israel; 3 Ministry of Health, Jerusalem, Israel; 4 Josiah Macy Jr. Foundation, New York, USA

**Keywords:** Innovation, Journal, Israel, Health policy

## Abstract

The Israel Journal of Health Policy Research (IJHPR) is a peer-reviewed, on-line, open access journal, sponsored by Israel’s National Institute for Health Policy Research. We believe that it is both an innovative platform and a platform for innovation. Within just 2 years of its launch in 2012, the IJHPR was accepted into the prestigious Web of Science – primarily because of its innovative positioning as a journal that is simultaneously national and international. This positioning has contributed to annual growth of over 20% in both submissions and publications and to the IJHPR being ranked among the top half of public health journals, just 6 years after its launch date.

The IJHPR has also served as a platform for numerous innovations, including:

- Sharing with the international community information about Israeli innovations in public health, health policy, health care delivery, and more.
- Enhancing the impact of empirical studies by Israeli scholars via commentaries by leading scholars from abroad – including 18 commentaries from scholars based at Harvard and one commentary by a Nobel laureate in economics.
- Developing a new genre of articles for Israel, namely, broad policy analyses focused on major challenges facing Israeli health care.
- Creating dynamic, constantly growing, article collections in such fields as digital health, pharmaceutical policy and health care equity, to highlight areas of excellence as well as important issues in Israeli health care and health policy.
- Disseminating to a wide audience the essence of major Israeli health policy workshops and conferences.

We feel that the IJHPR has significant potential to contribute more, and in new ways, in the years ahead. We look forward to your suggestions for innovative enhancements of the IJHPR.

## Background

**Fig. 1:**
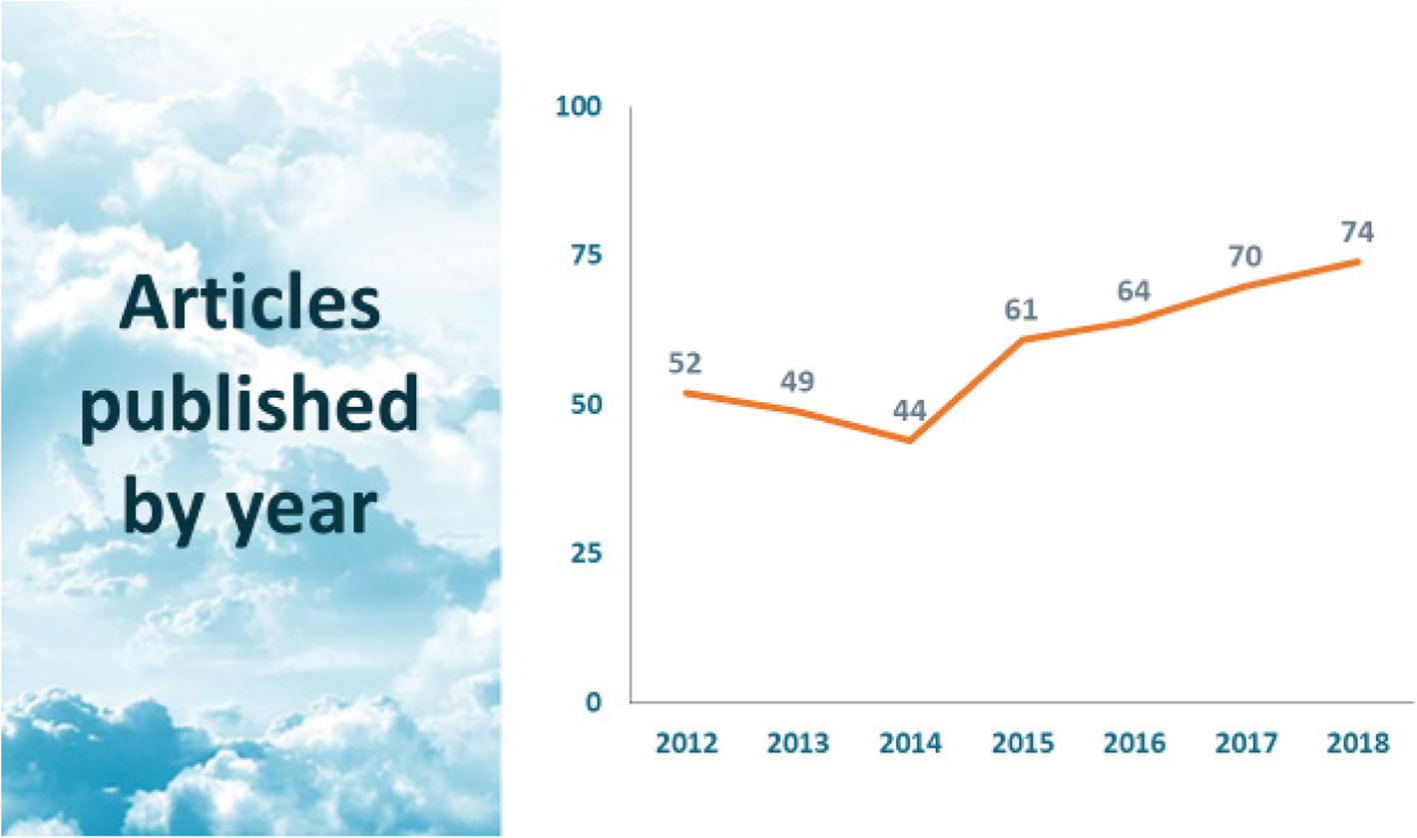
Articles published by year, 2012-2018

The Israel Journal of Health Policy Research (IJHPR) was launched in 2012 as an on-line, open access journal. It is sponsored by the Israel National Institute for Health Policy Research (NIHPR) and is published by BioMed Central. The number of articles published per year is increasing steadily (Fig. 1), and to date the journal has published almost 500 articles. The journal has a 2018 Web of Science impact factor of 1.66; and, just 6 years after being launched, it is ranked in the top half of journals in the Web of Science’s public health category.

This editorial is based on a presentation made at the NIHPR’s 7th International Jerusalem Conference on Health Policy, whose theme was “Health and Healthcare in the Age of Innovation” [7]. This editorial explores how the IJHPR is both an innovative platform and a platform for innovation; it makes use of the Oxford English Dictionary’s definition of innovation as “a new method, idea, product, etc.”. Our objectives in publishing this editorial include sharing the journal’s accomplishments with its authors and readers, clarifying for them the journal’s directions, encouraging strong submissions, and asking for input on ways in which the journal can continue to be innovative.

### The IJHPR as an innovative platform

The IJHPR is simultaneously a national journal and an international journal. Many journals, such as Health Policy and the International Journal on Equity in Health Care are fundamentally international in nature. Others, such as the Canadian Health Policy Journal or Health Affairs have a clear focus on a particular country with, perhaps, an occasional article about another country. The IJHPR is uncommon in that it seeks to inform the development of health policy both within a specific country (Israel) and globally.^1^ According to Thomson-Reuters (personal communication), this dual nature was one of the primary factors in its 2013 decision to include the IJHPR in its Web of Science database in the IJHPR’s second year of existence – an unusual step by this prestigious bibliographic database. The journal’s innovative positioning has also contributed to annual growth of over 20% in both submissions and publications.

**Fig. 2:**
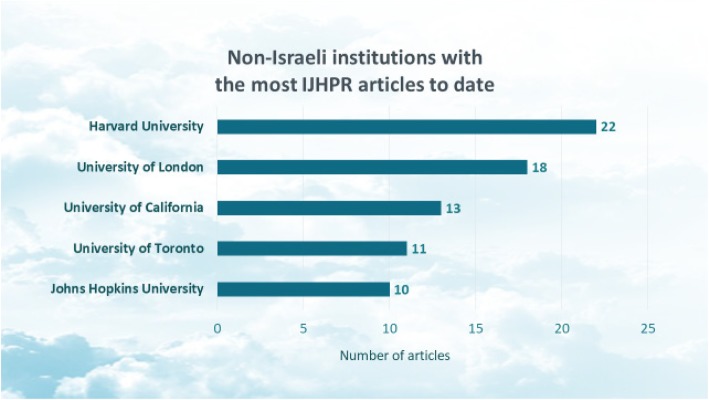
Non-Israeli institutions with the most IJHPR articles to date

In light of its dual nature,^2^ it is not surprising that about one-half of the articles published to date have included at least one author from a non-Israeli institution.^3^ Figure 2 highlights the non-Israeli institutions that are associated with the most IJHPR articles. These include institutions from the US, UK, and Canada.

**Fig. 3:**
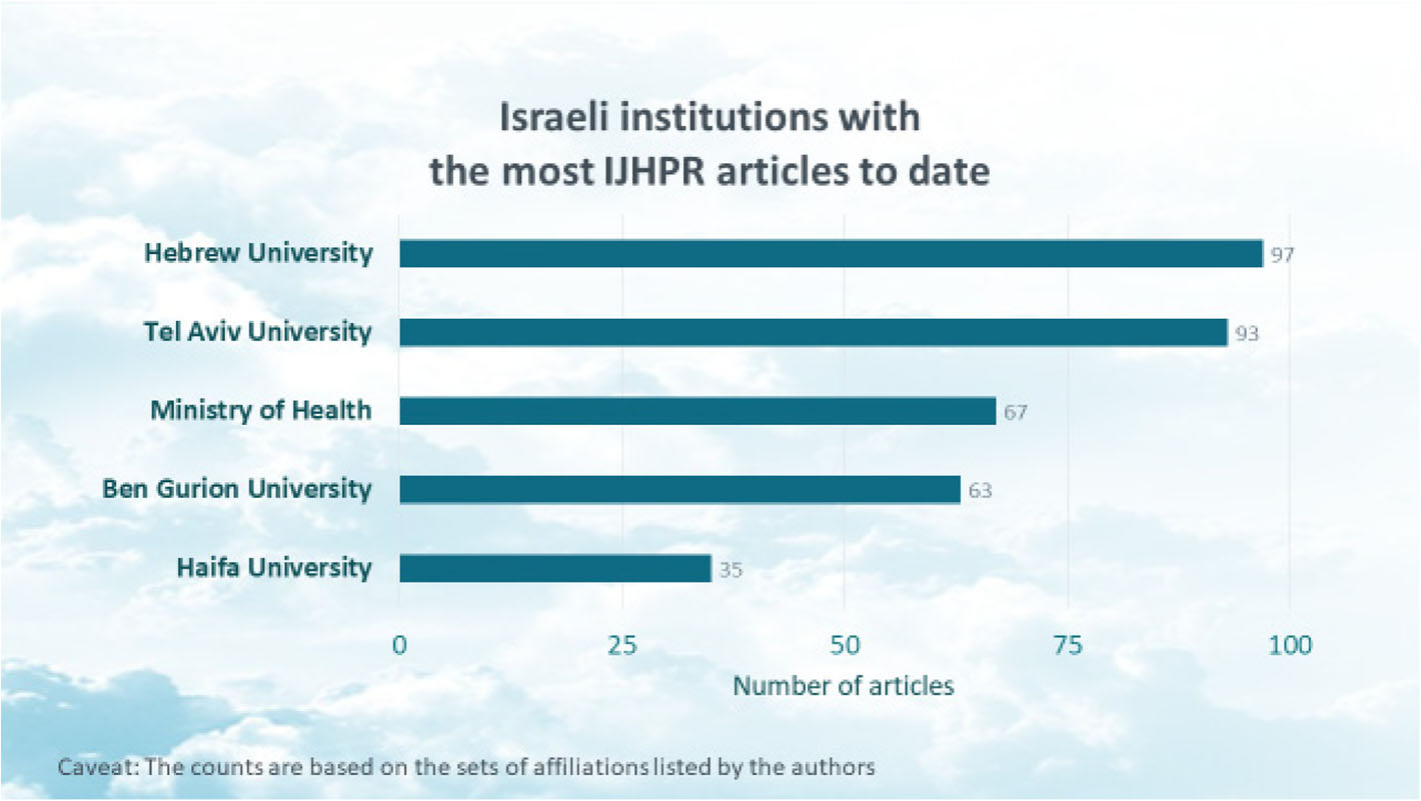
Israeli institutions with the most IJHPR articles to date

Figure 3 highlights the Israeli institutions associated with the most IJHPR articles.^4^ The fact that authors from the Ministry of Health have been frequent contributors is particularly noteworthy, and reflects the journal’s strong policy orientation. A broader analysis, not reflected in the chart, found that the IJHPR had a significant volume of contributions from virtually all of the Israeli universities, research centers, hospitals, health plans, and government agencies involved in health care.

### The IJHPR as a platform for innovation and for the dissemination of innovations

In addition to being an innovative platform, the IJHPR also serves in several ways as a platform for innovation and for the dissemination of innovations. As such, the journal continues to be a platform for innovations in writing and publishing that were not envisioned at the time of the journal’s establishment.

It is an excellent platform for sharing with the international community Israeli innovations in health policy and health care. For example, in 2012, a group based at the Hebrew University shared some of the accomplishments of Israel’s innovative system for monitoring quality of care in community-based health services [8]. Two years later, a group based at the Ministry of Health and the Gertner Center, shared an innovative Israeli approach to grappling with the ethical challenges posed by an important technological advance – the ability to use pre-implantation genetic diagnoses as a tool for sex selection [10]. And, in 2017, the IJHPR published an article from a group based at the Ministry of Health about how Israel is addressing the challenge of drug shortages – an issue relevant to many countries around the world [15].

The IJHPR is also being used to bring international perspectives to bear on important health system developments in Israel. After publishing empirical studies about an Israeli health care phenomenon or development, the editors often turn to international leaders in the relevant field and invite them to write a commentary on the Israeli study. For example, one of our commentaries was written by Alvin Roth, a Nobel laureate in economics. His commentary related to an Israeli paper that proposed a new way to assign young physicians to internships [13].

Oftentimes the commentaries are used to highlight the international significance of an Israeli study. This was the case, for example, in a commentary by Daniel Cotlear, the manager of the World Bank’s Universal Health Coverage Support Program [4]. Cotlear had read an IJHPR integrative article by two senior professionals from the Israel Ministry of Health analyzing Israel’s strategy for reducing health disparities [5], and felt that the Israeli experience could be very relevant and informative for a wide range of middle-income countries.

At other times, commentators use the international experience to suggest possible next steps for Israel – either in terms of program/policy development or in terms of research. For example, one of our commentators used her experience as an executive at the Visiting Nurse Service of New York to recommend potential strategies that Israel might adopt to provide a stronger support system for home care workers, more fully integrate them into the care team, and improve the occupational health and safety of this diverse, rapidly expanding workforce [14]. Similarly, an organizational consultant based in Toronto, writing a commentary on an Israeli paper about patient-initiated violence in hospitals, noted that Israel also need to address passive-aggressive and other low-level rude behaviors that take place frequently amongst hospital personnel [1].

The IJHPR has also encouraged a growing number of Israeli scholars to analyze and write about policy development in Israel. The topics have included tobacco control policy [12], policy related to drug use among Israeli backpackers [3], and efforts to reduce health disparities [5]. We believe it is important that such analyses be done and be published. Yet, if not for the existence of the IJHPR, it is unlikely that English language analyses of this sort would have been written; and even if they had been written, it would have been difficult to get them published in other peer-reviewed journals due to their focus on a specific, relatively small, country. Nonetheless, they contribute greatly to policy development in Israel and also contribute to comparative cross-national analyses. Thus, we see encouragement of these analyses and their publication as both innovative and important.

**Fig. 4:**
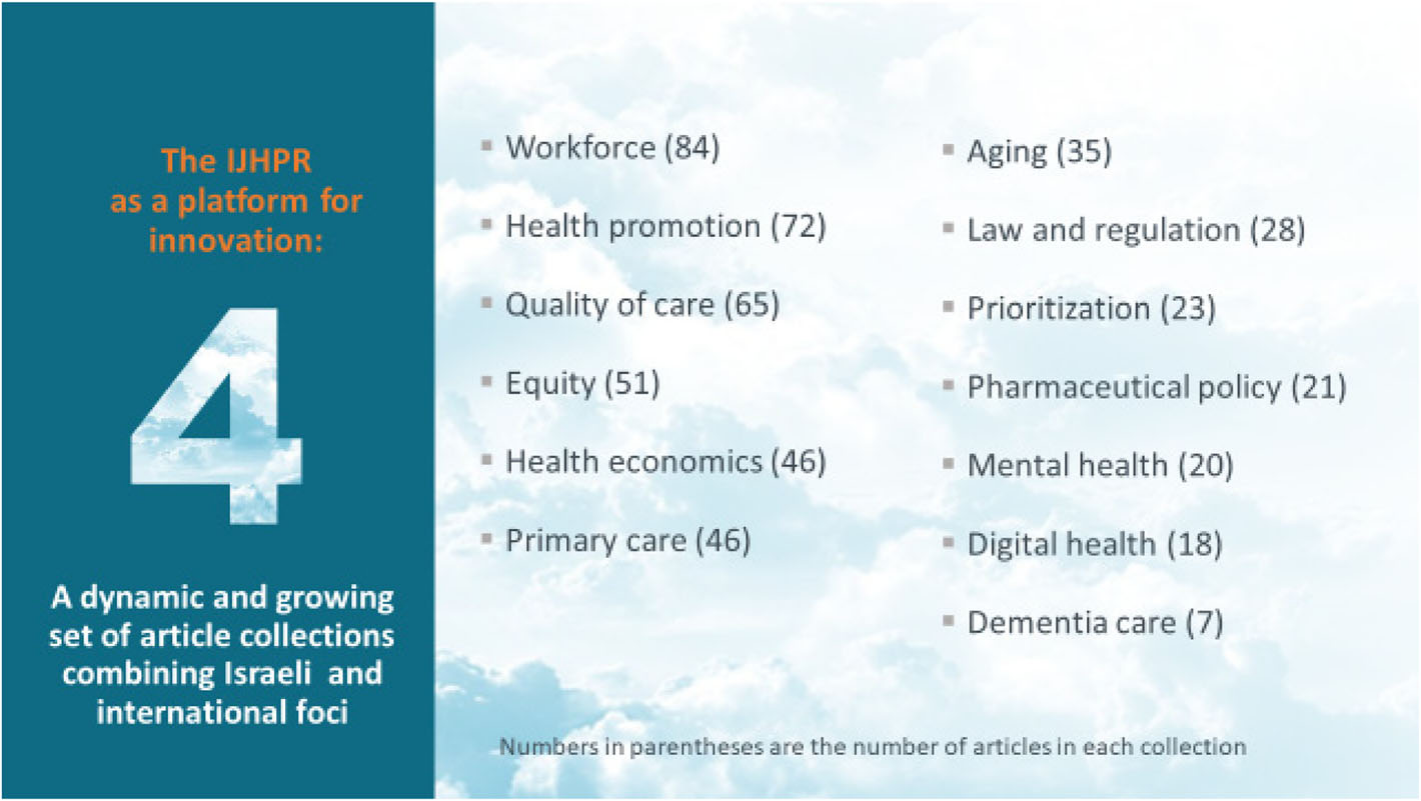
A dynamic and growing set of article collections combining Israeli and international foci

The IJHPR has also developed a large and growing set of article collections which bring together Israeli and non-Israeli writings on a specific topic.^5^ Our oldest and largest collections deal with the healthcare workforce (over 80 articles) and health promotion/disease prevention (over 70 articles). The newer collections include digital health, mental health, and pharmaceutical policy. The full list of collections can be found in Fig. 4. The on-line nature of the journal makes it easy to launch a collection with just a limited number of articles and then expand it over time. These collections are being used extensively for teaching purposes and for the writing and reviewing of grant proposals and journal articles.

The IJHPR also facilitates wide dissemination of key messages for health policy workshops and conferences held in Israel. These publications typically take the form of meeting reports, as has been the case for workshops on private health insurance [2], professionalism in the practice of medicine [9], and the potential role of the Holocaust in physician education [11]. Within less than 2 weeks after the IJHPR published the third mentioned report, it had been picked up by Richard Horton, the editor of “The Lancet,” one of the most widely read medical journals in the world, who wrote a commentary about it for his journal, entitled, “Medicine and the Holocaust—it’s time to teach” [6].

Another publication format related to meetings is publication of a supplement with the abstracts from the papers presented at a conference. The IJHPR did this recently with regard to the Jerusalem International Health Policy Conference on health care innovation [7].

The IJHPR is also an important and innovative vehicle of communication among Israeli researchers and between Israeli researchers and Israeli policymakers. As such, it facilitates new types of interactions and collaborations among them.

Finally, we are extremely proud that the IJHPR has published approximately 50 papers involving collaborations between an author based primarily at an Israeli institution and an author based at a non-Israeli institution. One such paper compared Israeli and Austrian regulation of egg cell donations [16]. It is by far the journal’s most downloaded article. We consider collaborative articles to be very important and plan to publish more of them in the months and years ahead.

## Conclusions

We have demonstrated several ways in which the IJHPR is an innovative platform and a platform for innovation. We also feel that the IJHPR has significant potential to contribute more, and in new ways, in the years ahead. These new ways could include, but are not limited to, those that take advantage of technological developments in such fields as artificial intelligence, applications, and social networking. We would appreciate your suggestions for additional ways in which the IJHPR can develop and innovate. Please send your ideas to us at ijhpr2@gmail.com.

## Data Availability

Data will be shared upon request.

## Footnotes

1 Interestingly, among the 82 journals in the Health Policy and Services category of the 2019 Journal Citation Report, only 6 included in the journal title the name of a specific country, and 4 others included in the journal title the name of a specific region or continent.

2 Israelis are very open and curious to learn from other countries’ experiences, and also want be keep up to date on what is going on in Israel, so this dual nature meets both needs in a single journal and is a good fit for Israel.

3 Almost half of the IJHPR articles (234 out of 478) published as of September 8, 2019 included at least one author with an affiliation with an institution in a country other than Israel. Most of the 234 were commentaries, while 64 of them were original research articles or integrative articles.

4 Note that these data rely on the affiliations listed by the authors themselves and these may not be complete. While authors are allowed to list more than one affiliation, and all listed affiliations are counted, it may be that some authors inadvertently neglect to list all of their institutional affiliations.

5 While persons interested in identifying a set of Israeli articles on a specific topic could use Google Scholar or Web of Science to do so, our article collections have two specific advantages. First, they are carefully curated by the editors, who are familiar with the content of the articles beyond the use of certain keywords. Second, they enable the editors to proactively alert scholars in a particular field that the IJHPR is interested in their field and has a collection of articles that could interest them.
